# Sarcoid-like reaction and hypothyroidism induced by PD-1 inhibitor treatment in metastatic renal cell carcinoma: a case report and literature review

**DOI:** 10.1186/s12890-024-02943-9

**Published:** 2024-03-08

**Authors:** Oadi N. Shrateh, Yazan Abugharbieh, Yousef Abu Asbeh, Hani hour, Iyad Awad, Sami bannoura

**Affiliations:** 1 Faculty of Medicine, Al-Quds University, Jerusalem, Palestine; 2 Deparment of Radiology, Al-Ahli Hospital, Hebron, Palestine; 3 Department of Thoracic Surgery, Al-Ahli Hospital, Hebron, Palestine; 4 Department of Oncology, Al-Ahli Hospital, Hebron, Palestine; 5 Department of Pathology, Al-Ahli Hospital, Hebron, Palestine

**Keywords:** Renal cell carcinoma, Pembrolizumab, Sarcoid like reaction, Hypothyroidism

## Abstract

**Background:**

Pembrolizumab is among the approved treatments for a variety of cancer types, including clear cell renal cell carcinoma (ccRCC). It has contributed to enhancing the prognosis of renal cell carcinoma. However, it is essential to be aware of the numerous potential immune-related side effects associated with its use.

**Case presentation:**

A 69-year-old patient with a history of metastatic renal cell carcinoma has been undergoing treatment with Pembrolizumab, an immune checkpoint inhibitor. The medication has led to the development of a sarcoid-like reaction, initially misinterpreted as cancer recurrence and progression. Additionally, the patient has experienced new-onset hypothyroidism, which has been attributed to the immunotherapy.

**Conclusion:**

Clinicians, including oncologists, endocrinologists, and radiologists, should maintain a high level of suspicions and awareness regarding the potential adverse events associated with newly introduced immunotherapies like pembrolizumab. This knowledge is crucial for the accurate diagnosis and appropriate management of patients receiving these treatments.

## Background

Renal cell carcinomas (RCCs) originating in the renal cortex account for approximately 80% of all primary renal tumors [1]. The global incidence of RCC exhibits variability, with roughly 400,000 new cases reported annually, resulting in approximately 170,000 fatalities [2]. RCC originates from the proximal tubule and typically exhibits a 3p chromosome deletion [3]. A notable correlation exists between a high nuclear grade and an unfavorable prognosis [4]. Advancements in curative surgery have led to improved case fatality rates, and early detection of smaller kidney tumors has boosted the 5-year survival rate, reaching approximately 76% (2009–2015) [5, 6]. In non-metastatic cases, radical nephrectomy remains the standard of care, proving particularly curative in early-stage cases [7].

Pembrolizumab, an immune checkpoint inhibitor (ICPI), gained approval as an adjuvant treatment for individuals who have undergone nephrectomy for clear cell renal cell carcinoma (ccRCC) [8]. Immunotherapeutic agents like pembrolizumab have revolutionized the management of metastatic diseases, demonstrating encouraging outcomes. Currently, they are being employed in the treatment of advanced melanoma, non-small cell carcinoma, and various solid tumors such as RCC [9]. ICPIs can cause some rare or very rare immune-related adverse events (irAEs) including cardiotoxicities, hematological toxicities, infection reactivations, and neurologic toxicities [10–13]. The most frequently encountered irAEs associated with pembrolizumab include musculoskeletal pain, fatigue, rash, diarrhea, pruritus, and hypothyroidism, occurring in approximately 20% of patients receiving this treatment [14]. In addition, there have been a few reported cases of pembrolizumab-induced sarcoid-like reactions [15–17].

However, the co-existence of two or more of pembrolizumab-induced adverse events in treatment of RCC is extremely rare. Herein, we report a case of sarcoid-like reaction concomitant with hypothyroidism induced by the ICPI during the treatment of metastatic renal cell carcinoma. highlighting the importance for radiologists and endocrinologists to recognize these autoimmune reactions when reporting treatment response to immunotherapy for metastatic RCC.

## Case presentation

**Fig. 1:**
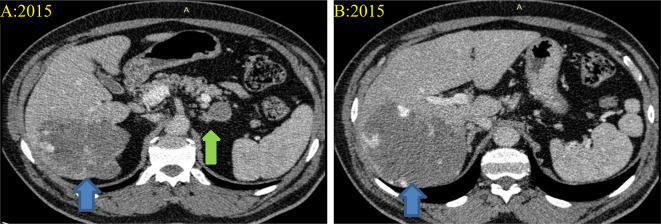
Computed Tomography (CT) scans at the level of the of the liver and adrenal glands (**A** & **B**) which reveal a large liver lesion with peripheral nodular enhancement and matching of the blood pool measuring about 5 × 7.5 cm compatible with his known hemangioma (**Blue arrows**). Left adrenal lesion of about 3 × 3 cm with an average density (0 HU) consistent with adrenal adenoma (**Green arrow**)

**Fig. 2:**
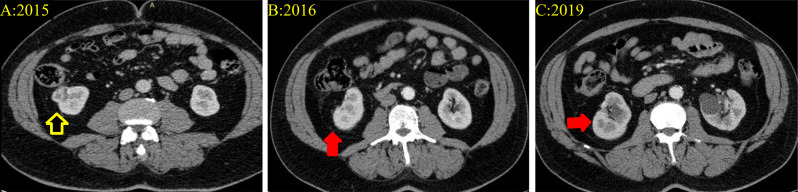
Selected CT scans at different times in which (**A**) is showing an exophytic rounded hyperenhancing cortical lesion in the lower pole of the right kidney measuring about 2.7 × 2.2 × 2.1 cm in in AP x CC x TS diameters respectively (**black arrow with yellow highlight**) consistent with the partially resected renal cell carcinoma of clear cell subtype. (**B** & **C**) demonstrating the site of Partial nephrectomy with clear surgical site (**Red arrows**)

A 69-year-old male patient, who had been under observation for liver hemangioma, underwent a computed tomography (CT) scan to explore the possibility of embolization treatment. During this evaluation, an incidentally discovered enhancing mass in the right kidney was identified and later confirmed to be renal cell carcinoma of clear cell type measuring 2.7 × 2.2 × 2.1 with T1a stage (refer to Figs. 1 and 2**).** In 2016, the patient underwent a partial nephrectomy with no reported complications, complaints, adverse events, or re-admissions. The patient has a medical history of diabetes mellitus, hypertension, seasonal allergies, and eczema. Notably, his father and sister succumbed to lung cancer, and his brother is currently being treated for leukemia. The patient’s past surgical history is unremarkable.

**Fig. 3:**
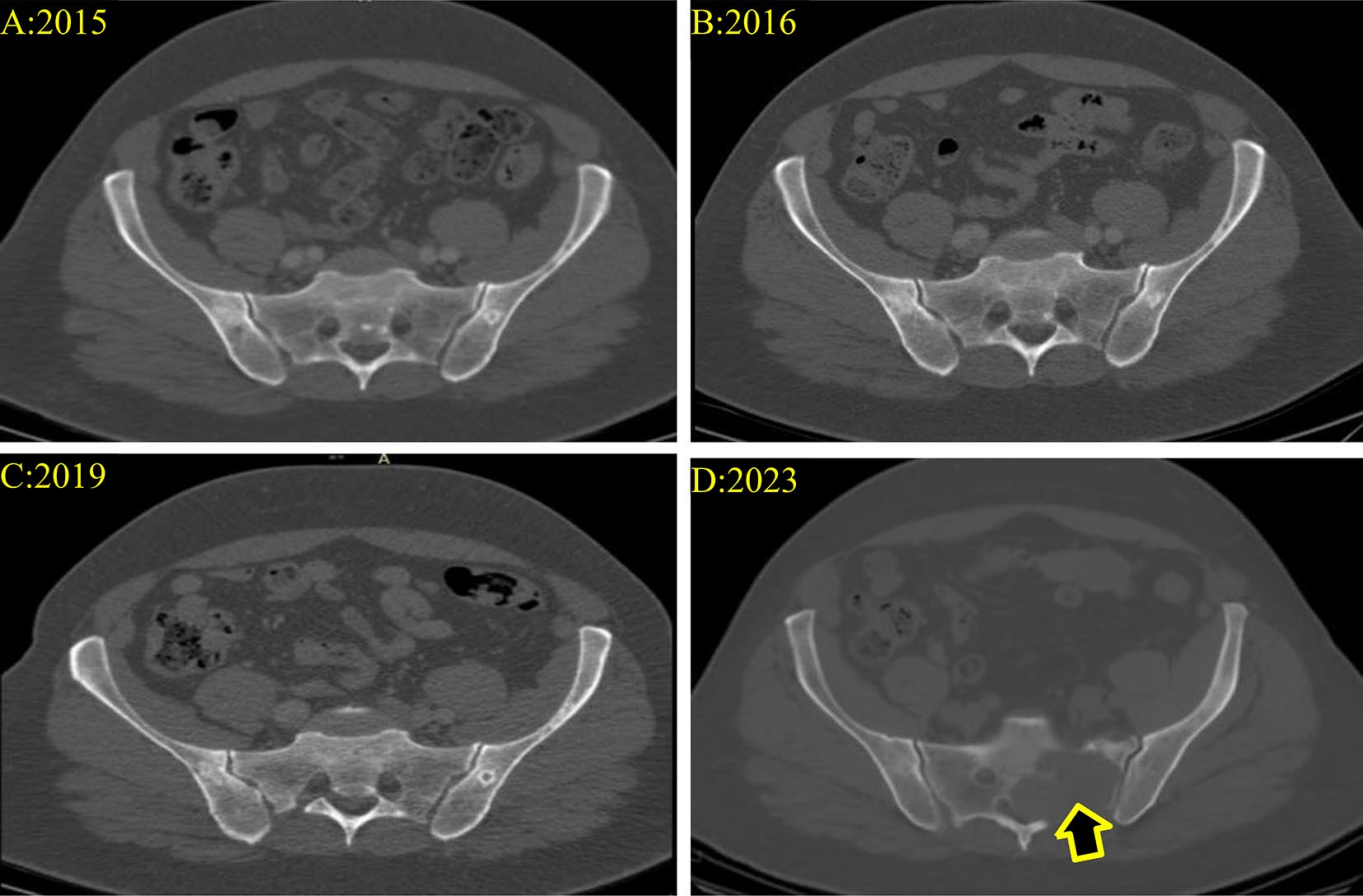
Selected Pelvic CT scans at variable periods interval which reveal normal pelvis CT scan without any suspicious lytic bony lesion (**A, B, C**). while in 2023 the scan showed an expansile, lobulated osteolytic destructive bony lesion in the left upper sacrum measuring about 5.8 × 6 × 6.2 cm in AP x CC x TS diameters respectively highly suspicious for serious malignant nature (**D**) (**The arrow**)

The patient underwent thorough monitoring through serial imaging, encompassing CT scans, and routine blood examinations until 2023. Throughout this duration, all imaging studies and blood tests consistently revealed no noteworthy abnormalities or indications of metastasis. Notably, there were no signs suggestive of potential sacral metastases development, as illustrated in Fig. 3.

**Fig. 4:**
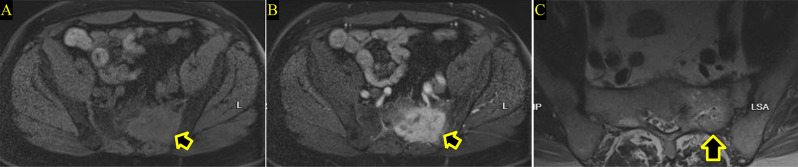
Magnetic resonance imaging (MRI) performed in 2023 with T1 precontrast (**A**), T1 Post-gadolinium (**B**) and T2 sequence (**C**) showing a left sacral mass lesion which exhibit low T1, high T2 signal with marked enhancement in keeping with hypervascular bony lesion (**Arrows**)

**Fig. 5:**
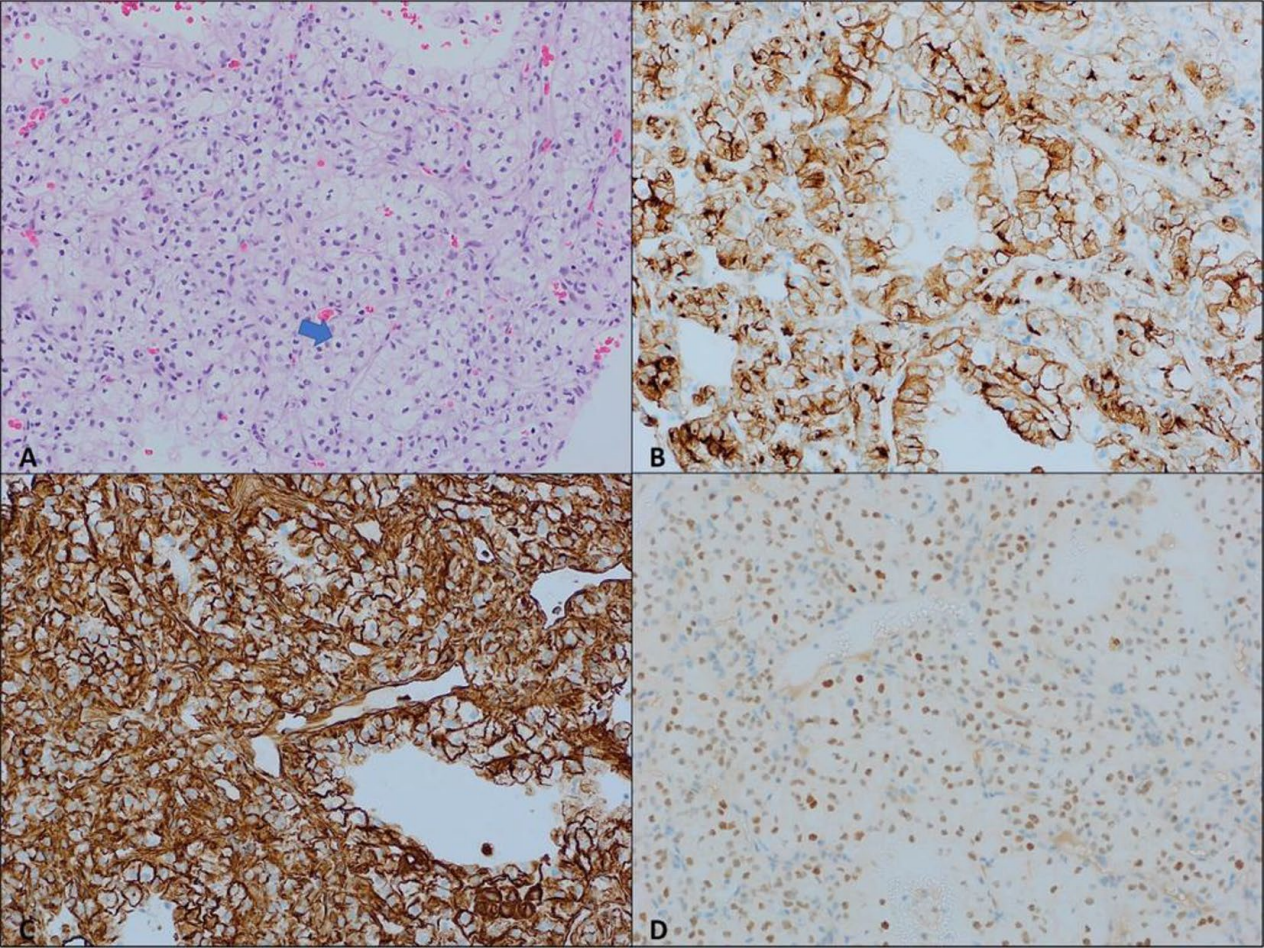
Clear cell renal cell carcinoma; (**A**) Sections show compact nests of tumor cells with abundant clear cytoplasm and distinct cytoplasmic membrane (arrow) (H&E, 20X). (**B**–**D**) The tumor cells are positive for CD10, vimentin, and Pax8 immunostains, respectively confirming the diagnosis. (20X)

In February 2023, the patient began encountering pain in the left hip, prompting a medical consultation. Initially, physical therapy was recommended, resulting in modest improvement. However, with the progression of symptoms, further investigation became necessary. A magnetic resonance imaging (MRI) was conducted, revealing a suspicious solitary lytic destructive bony lesion, as depicted in Fig. 4. Subsequently, a biopsy was undertaken, and the histopathology report confirmed the lesion’s origin as metastatic clear cell renal cell carcinoma (RCC), as shown in Fig. 5. A positron emission tomography (PET) scan was performed, revealing no evidence of hypermetabolic active lesions, with the exception of the left sacral tumor. This outcome implies that, aside from the known sacral tumor, no other areas exhibited significant metabolic activity indicative of active cancer cells throughout the body.

**Fig. 6:**
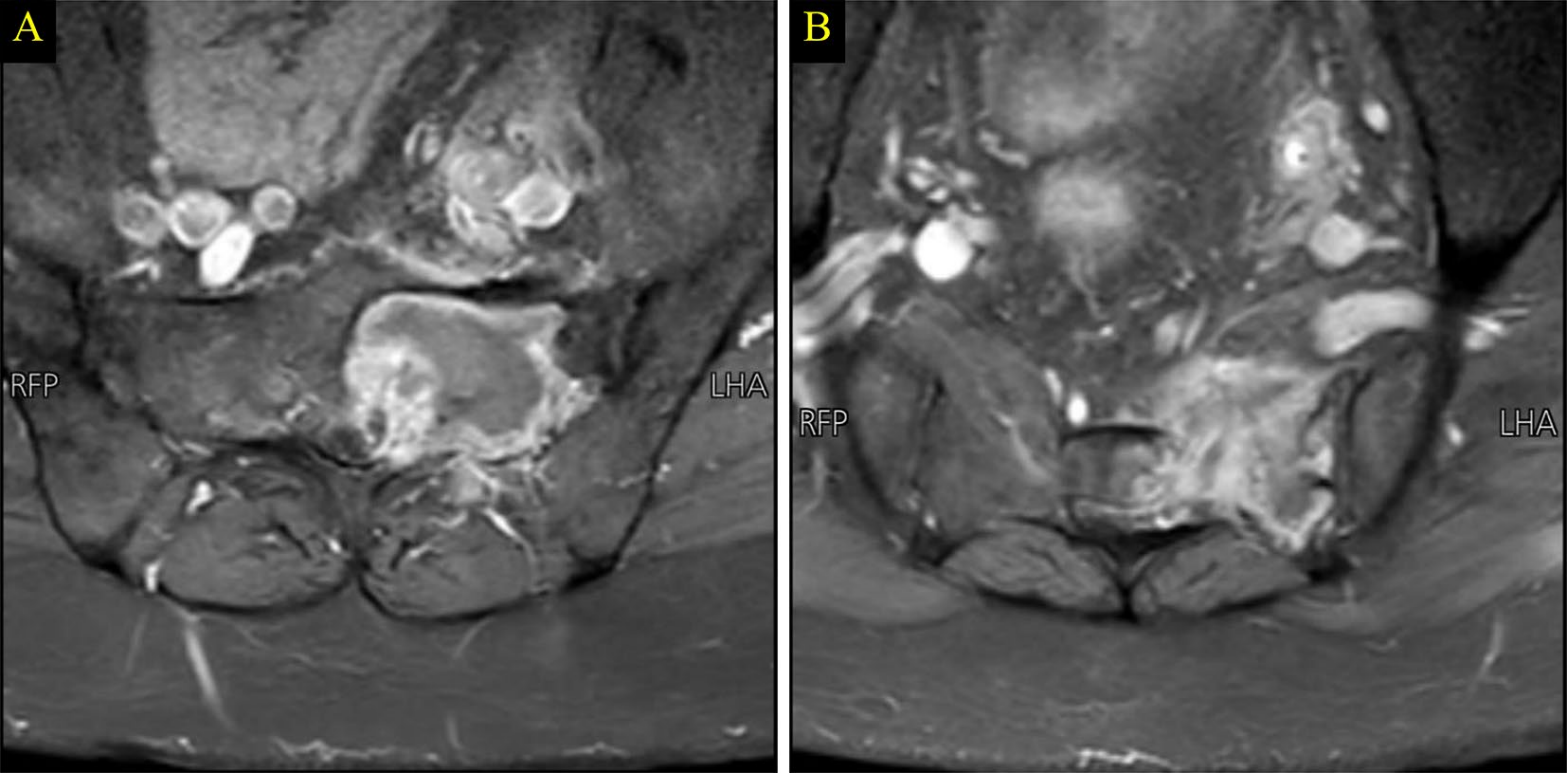
Multilevel axial T1 MRI post- contrast sequences reveal a large necrosis in the site of bony metastatic lesion indicating good response to the treatment

The patient’s treatment regimen involves the administration of axitinib. Initially, he commenced with a dosage of 1 tablet 5 milligrams (mg) twice daily. After 9 weeks of treatment, the dosage was adjusted, and he transitioned to 1 tablet once daily. This modification was guided by the patient’s response to the treatment and aimed at minimizing potential side effects or tolerability issues that may arise during the course of treatment. Alongside axitinib, the patient is also receiving pembrolizumab intravenously at a dose of 200 mg every 3 weeks. The patient undergoes regular weekly follow-up visits, revealing notable improvement in the clinical course. Radiologically, MRI indicated significant necrosis at the site of the sacral metastatic lesion, affirming a positive response to the treatment (refer to Fig. 6). Notably, no abnormalities were observed in the whole-spine MRI.

**Fig. 7:**
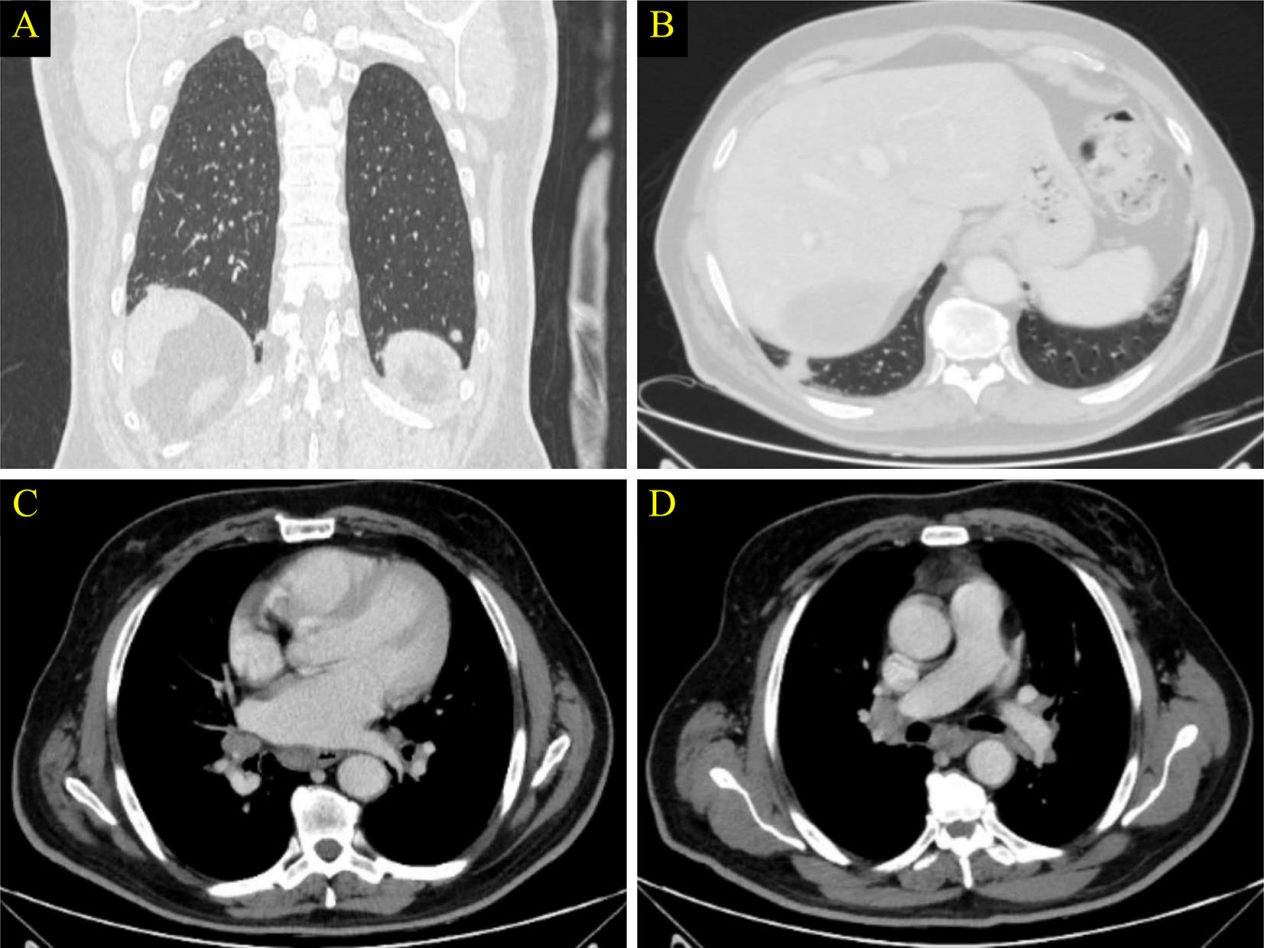
Selected coronal (**A**) and axial (**B**) cuts of CT scan with lung window demonstrating multiple bilateral subpleural lung nodules. (**C** and **D**) are selected axial CT scan at the level of the mediastinum showing multiple mediastinal and hilar lymph nodes (**arrows**)

**Fig. 8:**
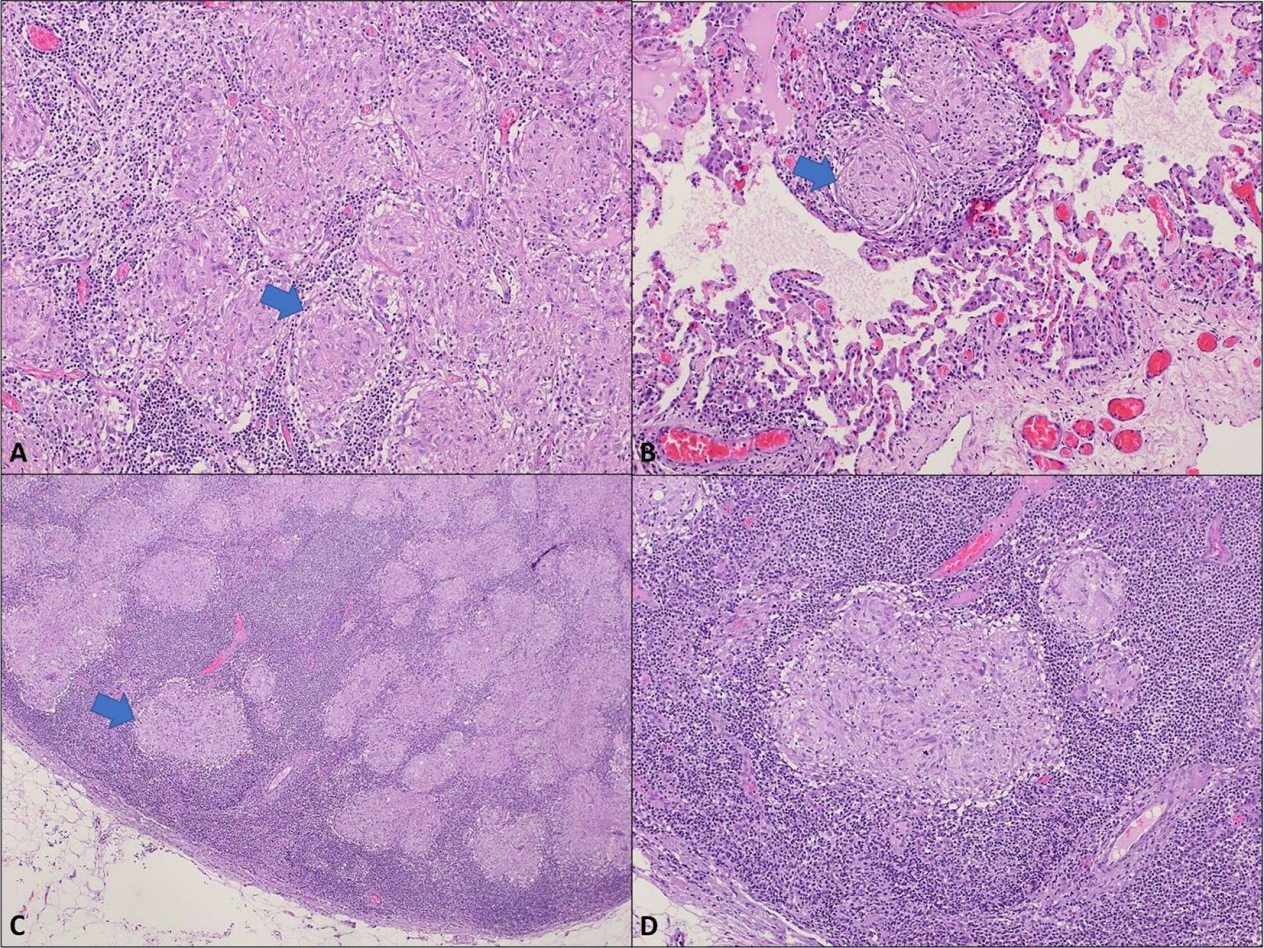
Non-necrotizing epithelioid granulomas; **A**, **B**. Section of the lung nodule shows numerous non-necrotizing epithelioid granulomas (**Arrows**); alveolar lung parenchyma is seen in lower half of picture **“B”** (**H&**;**E**, **10X**). **C**, **D**. The submitted mediastinal lymph node shows similar finding of numerous non-necrotizing epithelioid granulomas. (**Arrow**) (**H&**;**E**, **4X**, **20X**). Special stains (not shown) including PAS and acid fast are negative for fungal microorganisms and mycobacteria, respectively. There is no evidence of malignancy

During the next annual follow-op visit, radiological imaging showed multiple bilateral subpleural lung nodules as well as multiple mediastinal and hilar lymphadenopathy (Fig. 7). It’s worth to note that, these abnormalities were not present at the previous imaging. Based on these findings, the patient was suspected to have a metastatic lung cancer and treatment line was planned to be changed. However, a multidisciplinary discussion was conducted and sarcoidosis-like reaction as a side effect of patient’s regimen was came to our differential diagnosis. Therefore, complete workup was performed and detected a markedly elevated serum levels of thyroid stimulating hormone (TSH) of 18 uIU/mL, low normal free T4 and the absence of thyroid peroxidase (TPO), angiotensin converting enzyme (ACE) of 64.40 U/L. The histopathologic results of tow biopsies from the lung nodules and mediastinal lymph nodes demonstrated non-necrotizing epithelioid granulomas consistent with a sarcoid-like reaction (Fig. 8). Thyroxine 100 mg and symptomatic treatment for cough were initiated. The corticosteroid was not given because it may compromise the immune response of the patient. The patient was scheduled regular follow-up visits for a duration of 8 months with improvement of his clinical status.

## Discussion

Partial or radical nephrectomy stands as the established standard of care for treating locoregional clear-cell renal-cell carcinoma [18, 19]. However, it’s important to note that nearly half of these patients will eventually experience disease recurrence post-surgery, with a significant portion of them developing distant metastases, which significantly shortens their life expectancy [20]. Risk factors contributing to disease recurrence and reduced recurrence- and metastasis-free survival include disease stage, tumor size, nuclear grade, and regional lymph-node involvement of the resected tumor [19, 21–25]. In selected cases of advanced renal-cell carcinoma (M1 stage, indicating metastasis in distant organs or tissues), surgery plays a role in the management of patients with surgically resectable oligometastatic sites. Following nephrectomy and metastasectomy, these patients are categorized under “M1 with no evidence of disease” (M1 NED), defined as the resection of the primary tumor and isolated, solid, soft-tissue metastases. It’s important to highlight that even patients with M1 NED status are at high risk for disease recurrence [22, 26].

For patients with renal-cell carcinoma post-surgery, there is presently no universally accepted standard adjuvant therapy with robust supporting evidence. Current treatment guidelines suggest participation in a clinical trial or opting for active surveillance following surgical intervention [18, 19]. Anti–programmed death 1 (PD-1) antibodies like pembrolizumab have demonstrated efficacy both as standalone treatment and in combination with other agents in patients diagnosed with advanced renal-cell carcinoma [27–36]. As a result, these antibodies hold promise as a potential adjuvant approach for managing this disease. The use of immune checkpoint inhibitors, such as pembrolizumab, has now become an essential element of the oncological treatment toolbox. Nevertheless, due to the relatively recent introduction of these therapies, the precise mechanisms underlying the development of immune-related adverse events remain not fully elucidated. Furthermore, there is a lack of clarity regarding whether the occurrence of a single immune-related adverse side effect makes patients more susceptible to subsequent unwanted side effects throughout their clinical course.

Immune checkpoint inhibitors (ICIs) represent a prominent class of monoclonal antibodies extensively utilized in the treatment of a diverse range of malignancies [37]. Programmed death protein 1 (PD-1) is expressed on activated T lymphocytes, recognizing programmed death ligands 1 and 2 (PDL-1 and PDL-2), which are commonly expressed by tumor cells to evade the immune system [38]. Pembrolizumab, a humanized immunoglobulin G4 (IgG4) monoclonal antibody, belongs to the category of immune checkpoint inhibitors (ICIs). It specifically targets programmed death protein 1 (PD-1), thereby interrupting the interaction between PD-1 and PDL-1 and PDL-2. This disruption leads to an enhancement of the T-cell mediated immune response against tumor cells [39].

It’s important to acknowledge that immune checkpoint inhibitors are not without their adverse effects, which encompass autoimmune toxicity stemming from the risk of activated T lymphocytes attacking healthy tissues. This phenomenon is commonly referred to as immune-related adverse events (irAEs) and can impact various organs and tissues, including the skin, gastrointestinal tract, liver, and endocrine system, among others [40]. Moreover, ICIs have been associated with a diverse range of neurologic immune-related adverse events (irAEs), such as meningoencephalitis, myasthenia gravis, and various neuropathies, in addition to affecting endocrine systems with outcomes like hypo- or hyperthyroidism [41–45].

The term “sarcoid-like reaction” typically denotes localized reactions, in contrast to the systemic process observed in sarcoidosis. However, systemic involvement affecting organs like the lungs, skin, and kidneys can also occur. A distinctive feature of sarcoid-like reactions is the presence of noncaseating granulomas [46]. It’s crucial to recognize that sarcoid-like reactions can sometimes mimic cancer worsening or recurrence. In such instances, a combination of clinical presentation, alongside a suitable biopsy, radiographic evidence indicating bilateral hilar lymphadenopathy (with paratracheal lymphadenopathy), and elevated serum ACE levels, can help differentiate sarcoid-like reactions from cancer recurrence or metastasis. In our patient’s case, the diagnosis of a sarcoid-like reaction wasn’t initially suspected, primarily due to its atypical presentation with the absence of clinical symptoms or skin lesions. However, after a multidisciplinary discussion and the identification of noncaseating granulomas in the biopsy, along with elevated ACE levels, the focus shifted away from cancer recurrence or metastasis. It’s important to note that sarcoid-like reactions are not exclusive to pembrolizumab but are also associated with other checkpoint inhibitors like ipilimumab and nivolumab [47]. Given the increasing use of these drugs as a standard of care for various malignancies, it’s imperative for both oncologists and radiologists to remain vigilant about the possibility of sarcoid-like reactions. Recognizing these reactions is crucial for appropriate management and for potentially assessing therapeutic response.

In their study [48], Gaibor C, and his colleagues highlighted the simultaneous occurrence of several immune-related adverse events in a patient undergoing treatment with pembrolizumab. Initially, the patient developed seronegative myasthenia gravis, which was diagnosed based on positive electromyography and was attributed to the use of pembrolizumab. Subsequently, during the same admission and in light of the patient’s history of multiple falls, a highly elevated thyroid-stimulating hormone (TSH) level was detected, along with inappropriately low normal free T4 and the absence of thyroid peroxidase (TPO) antibodies, ultimately leading to the diagnosis of pembrolizumab-induced hypothyroidism. our patient developed sarcoid-like reaction associated with hypothyroidism as a complication of pembrolizumab therapy.

Consequently, it is imperative to conduct additional research into the side effect profile of pembrolizumab, particularly in the context of autoimmune-related adverse effects and the frequency of their concurrent occurrence. This knowledge is crucial to ensure that clinicians remain informed about the potential untoward effects associated with these novel biological drugs. These findings will have a substantial impact on clinical management and therapeutic decision-making, benefiting both physicians and patients in the future.

In our case report, the patient exhibited a notable treatment response, which prompts us to consider potential predictors of such efficacy. Notably, the presence of a sarcoid-like reaction and concomitant hypothyroidism in the patient’s medical history may warrant attention as potential indicators of treatment response. Moreover, the coexistence of hypothyroidism with the observed treatment response adds complexity to our understanding. Hypothyroidism has been implicated in various immune dysregulations, potentially influencing treatment responsiveness through its effects on immune function. Exploring the interplay between thyroid function and treatment outcomes in patients with similar presentations may unveil novel avenues for optimizing therapeutic interventions.

While our findings are based on a single case and require validation in larger cohorts, they underscore the importance of considering sarcoid-like reactions and hypothyroidism as potential predictors of treatment response in similar clinical scenarios. Future research endeavors should prioritize elucidating the underlying mechanisms and clinical implications of these associations to enhance prognostication and therapeutic decision-making.

## Conclusion

Oncologists and radiologists should remain vigilant about the link between immunotherapy and the potential development of sarcoid-like reactions and/or hypothyroidism to prevent mistakenly attributing new imaging findings to disease progression. This awareness is particularly critical given the swift adoption of these drugs as the standard of care for various other types of tumors, including renal cancer. Consequently, radiological changes should always be assessed in the context of the specific immunotherapy agent (or chemotherapy agent), the response of the preexisting malignancy, and the patient’s symptoms. In cases of uncertainty, biopsies are often necessary to confirm the diagnosis and guide treatment decisions.

## Data Availability

No datasets were generated or analysed during the current study.

## Abbreviations

RCC: Renal cell carcinomas
ICPI: immune checkpoint inhibitor
ccRCC: clear cell renal cell carcinoma
irAEs: immune-related adverse events
PD-1: Anti–programmed death 1
TSH: thyroid-stimulating hormone
TPO: thyroid peroxidase
